# Novel Glycolipids Synthesized Using Plant Essential Oils and Their Application in Quorum Sensing Inhibition and as Antibiofilm Agents

**DOI:** 10.1155/2014/890709

**Published:** 2014-01-14

**Authors:** Ruchira Mukherji, Asmita Prabhune

**Affiliations:** Division of Biochemical Sciences, National Chemical Laboratory, Pune 411008, India

## Abstract

Essential oils (EOs) form an important part of traditional medicine so their anti-microbial and, in the recent past, antiquorum sensing activity has been well studied. However it is likely that due to their hydrophobic nature and reduced solubility in aqueous environments full potential of their activity cannot be realized. hence it is only rational to formulate a process to make these molecules more polar in nature. The present paper reports synthesis of sophorolipids using 12 different essential oils as substrates, thus providing surfactant-like properties to these EOs. The synthesis protocol makes the use of *Candida bombicola* ATCC 22214 as producer organism. The production process required 7 days of incubation at 28°C and 180 rpm. Preliminary characterization of the synthesized essential oil sophorolipids (EOSLs) was performed using thin layer chromatography (TLC) and Fourier transform infrared spectroscopy (FTIR). Additionally, essential oils that were incapable of mediating quorum sensing inhibition (QSI) on their own became potent quorum sensing inhibitors upon conversion into their corresponding EOSLs. Antibiofilm potential of these EOSLs was also demonstrated using *V. cholerae* as test organism. Use of essential oils as substrates for glycolipid synthesis has not been attempted previously, and hence this is the first report.

## 1. Introduction

Essential oils (EOs) have always garnered an important place in traditional medicine and amongst alternative healing practitioners. With their multitude of immunomodulatory and antimicrobial activities they have been used since many years in treatment of variety of conditions [1]. Essential oils are a mixture of numerous volatile components that are produced as a result of plant secondary metabolism. EO components can be differentiated in two different but biosynthetically related groups. The two main groups are compounds of terpene and terpenoid origin and the aromatic and aliphatic components [2]. EOs are extracted from aromatic and medicinal plants using a variety of different methods, including water or steam distillation of different plant parts.

Antibacterial activity of various EOs has been studied before by different groups around the world [3–6]. In the recent past quorum sensing inhibition mediated by essential oils and their components has also been analyzed [7–12]. Quorum sensing (QS) is the form of bacterial communication that allows individual bacterial cells to come together and function as a single entity protecting them from diverse deleterious conditions [13, 14]. A variety of genes are under the quorum sensing control regulon many of which modulate bacterial virulence including genes involved in exoenzyme production and biofilm formation [15–17]. In this light, inhibition of quorum sensing has been envisioned to be the new target for developing sustainable anti-infective therapies because impediment in QS will weaken the virulence of invading pathogens making them more susceptible to the applied mode of treatment [18].

Essential oil glycolipids have never been synthesized before. Rationale behind synthesizing such molecules was that, upon conversion into their corresponding SLs, the component essential oils may acquire some additional property which has been unforeseen previously and it might also increase their solubility in aqueous medium. This was indeed observed when some of the EOs reported in this study did not show QSI potential on their own but became potent QS inhibitors upon conversion into corresponding EOSLs. There are no reports that mention inhibition of quorum sensing by tea tree oil, bergamot oil, and basil oil and although the individual oils have no activity, after conversion into their corresponding SLs they have been shown be potent inhibitors of bacterial quorum sensing. The present report thus describes a method of preparation of these essential oil sophorolipids (EOSLs) which possess both QSI and biosurfactant like property. In addition all the synthesized EOSLs have also been shown to possess various degrees of anti-biofilm activity against *V. cholerae* biofilm. Moreover this is also the first report of use of Ylang ylang oil and Frankincense oil alone and its EOSL from for inhibition of QS mediated phenotypes.

## 2. Material and Methods

### 2.1. Culture Conditions and Maintenance of Microorganisms

*Candida bombicola* ATCC 22214 used for EOSL synthesis was maintained on MGYP agar slants (malt extract, 0.3 g%; glucose, 2 g%; yeast extract, 0.3 g%; peptone, 0.5 g%; and agar, 2.0 g%). The microorganism was subcultured every 4 weeks and maintained at 4°C in a refrigerator. *Chromobacterium violaceum* CV026 (kindly provided by Dr. Paul Williams, University of Nottingham) was grown in Luria Bertani broth supplemented with 100 *μ*g/mL ampicillin and 30 *μ*g/mL kanamycin. Culture was preserved in the form of glycerol stock and was revived whenever required. *Vibrio cholerae* MTCC0139 used in the anti-biofilm assays was grown in Luria Bertani broth without antibiotics and maintained in the form of glycerol stock kept at −70°C.

### 2.2. Inoculum Development and Production of EOSLs

10 mL of MGYP broth was inoculated with *Candida bombicola*. After 24 h incubation at 28°C, 180 rpm, it was added to 90 mL MGYP broth and was incubated further for another 48 h. After 48 hours of growth cells were harvested by centrifugation at 5000 rpm for 20 mins. The pellet was then redispersed in production medium of 10% glucose [19]. Substrates, that is, oleic acid and selected essential oils (purchased from Soulflower India Ltd.) in different ratios of ratios 1 : 20, 1 : 10, 1 : 5, and 1 : 2, were then added to the production medium and the flask was incubated at 28°C, 180 rpm for a period of 7 days. After the production period cells were removed by centrifugation at 5000 rpm for 20 mins. The culture supernatant containing the product was extracted thrice with equal volume of ethyl acetate. The aqueous layer was then separated from the solvent layer which contained the product. Rotary evaporation of the solvent layer yielded the synthesized EOSLs. The product was collected in a clean dry glass vial and purged to remove any traces of ethyl acetate. Different EOs gave different yields of resultant product.

### 2.3. TLC Analysis of the Synthesized EOSLs

New extracted and concentrated EOSLs were compared against previously synthesized OASL using thin layer chromatography (TLC). TLC was performed on commercially available silica gel coated aluminum sheets (Merck Aluminum TLC Silica Gel Plates 60 F 254). The solvent system used was 65 : 15 : 2 chloroform : methanol : water (v/v/v). The bands on the gel after completion of the run were visualized using iodine vapors. Yellowish brown bands appear on a white background after incubation with iodine vapors for a period of 3 minutes. The band migration pattern obtained with OASL was compared with each of the synthesized EOSL.

### 2.4. Oil Displacement Activity of Synthesized EOSLs

The oil displacement test is a method used to measure the diameter of the clear zone, which occurs after addition of a surfactant-containing solution to an oil-water interphase. The oil displacement test was done by adding 20 mL of distilled water and 3 mL of Jatropha oil to a 90 mm petri dish. 30 *μ*L of 10 mg/mL solution of synthesized EOSLs was dropped onto the oil-water interface. The diameter of the clear halo formed after displacement of oil was visualized under visible light and measured after 10 s.

### 2.5. FTIR Analysis of the EOSLs

FTIR spectroscopic analysis of the synthesized EOSLs and oleic acid sophorolipid (OASL) alone was performed to compare and analyze the similarities and differences in the newly formed EOSLs and original OASL. It was performed using Perkin Elmer FTIR system Spectrum BX over the spectral range of 400–4000 cm. EOSL samples were prepared by making a pellet in potassium bromide (KBr). Data from 17 consecutive scans was collected. Spectral data obtained was plotted on a graph of transmittance (%) versus wavenumber (cm^−1^).

### 2.6. Quorum Sensing Inhibition by EOSLs

Antiquorum sensing activity of essential oils alone, Oleic acid sophorolipid alone, and the newly synthesized EOSLs was performed using method described elsewhere with slight modifications. Briefly 50 *μ*L of overnight broth culture of CV026 was added to 5 mL of molten cooled Luria Bertani soft agar along with 0.25 *μ*L (corresponds to 1.25 *μ*M) of QS signal molecule, C6-HSL, and the mixture was overlaid onto Luria Bertani Agar plates. After the overlay solidified wells of 6 mm diameter were dug using a sterile corkborer. 50 *μ*L of 20 mg/mL solution of the synthesized EOSLs was added to the wells and the plates were incubated overnight at 28°C. Eos and EOSLs with capacity to inhibit QS showed a colorless zone around the well in a purple mat of violacein produced by test culture CV026 [20]. All experiments were carried out in triplicate for the sake of reproducibility.

### 2.7. Antibiofilm Activity of the Synthesized EOSLs

Biofilm formation is another phenotype governed by quorum sensing. Any compound/molecule capable of impeding this form of bacterial communication will also inturn disrupt bacterial ability to form biofilms. To observe anti-biofilm activity of EOSLs 10 *μ*L of overnight culture of *Vibrio cholerae* was added to 2 mL of sterile Luria Bertani medium in a 30 mm petri dish containing a sterile coverslip. 10 *μ*L of 10% EOSL solution was added to each test plate, respectively, and the plates were incubated at 30°C for 2, 4, 6, and 8 hours. Control plates without EOSLs served as system for monitoring un-interrupted biofilm formation by test organism *V. cholerae*. Biofilm formed after each incubation period was visualized using crystal violet staining method. Briefly, spent medium was discarded after completion of incubation period and the cover slips were rinsed twice with Milli-Q water to remove unadhered bacterial cells. The biofilm was then stained with 0.4% crystal violet solution for 5 mins, after which the staining solution was removed and the biofilm was gently washed twice with Milli-Q water and was allowed to air-dry [21]. Stained biofilm was then visualized under light microscope at 40x magnification.

## 3. Result and Discussion

Investigation into quorum sensing inhibition by essential oils obtained from different sources is underway in various laboratories and anti-QS property of various essential oils is coming to light. In our report anti-QS potential of 12 oils has been studied and the data has been presented in Table 1. Majority of oils used in this study, when tested alone, showed marginal QSI potential mostly because of their reduced solubility in the growth medium. Keeping this in mind the assay protocol was modified to include an emulsifying agent that could increase the solubility of oils in the growth medium, thus making them more effective than before. Oleic acid SL at a final concentration of 10 mg/mL (no inherent QSI activity of OASL at this concentration) helped emulsify the oils completely before addition to the agar wells in the test plate. In case of lemongrass oil + OASL, approximately 50% increase in zone of inhibition was observed and other oils that showed no QSI activity before became active when added in combination with OASL, namely, basil oil + OASL showed an 11% increase in zone size, ylang yalng oil + OASL 12% increase in activity, and peppermint oil + OASL 16% increase in QSI activity (Table 1). These observations led to the musing that if the EOs could be converted into their corresponding EOSLs they might acquire indigenous QS inhibition property.

**Figure 1:**
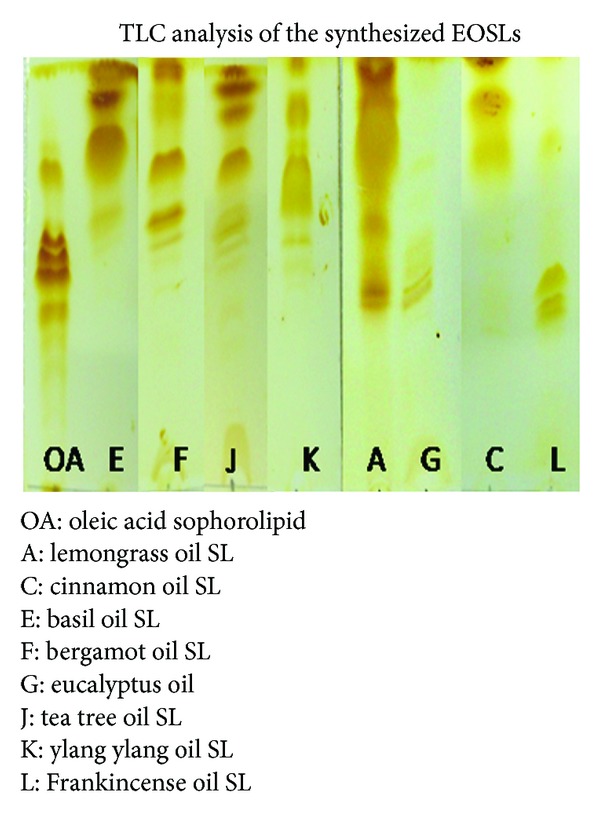
TLC analysis of synthesized EOSLs.

**Figure 2:**
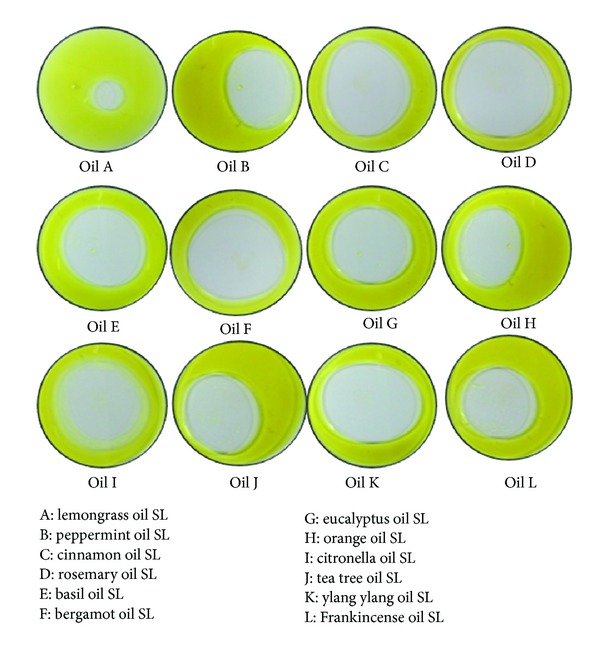
Oil displacement activity of the synthesized EOSLs.

For synthesizing these EOSLs, *Candida bombicola *ATCC 22214 was used as the producer organism. *C. bombicola* ATCC 22214 mediated SL synthesis protocol has already been well established in our group [19], but use of plant EOs as substrates for glycolipid synthesis has not been reported before. Initially EOs alone were added to the production medium (10% glucose) for SL synthesis but this led to partial cell death and after the 7-day incubation period there was very little accumulation of synthesized product, which was unrecoverable. Hence Oleic acid (OA) was used as an inducer molecule so that the organism adapts better to the newly added substrate and synthesis takes place more efficiently. It is hypothesized that SL allows easy emulsification of the added essential oil and thus aids in incorporation of the EO in final product. OA was used as an inducer in varying ratios of OA : EO, starting from low concentration of OA to progressively higher, namely, 1 : 20, 1 : 10, 1 : 5, and 1 : 2. However of all the ratio of OA : EO tested, 1 : 10 (OA : EO) was found to work well. All the synthesized EOSLs were analyzed by TLC and they were compared with an OASL control. All, except three, synthesized EOSLs showed bands differing from that of OASL molecule (Figure 1). Also, as expected, all the EOSLs were able to displace oil at the oil-water interface to varying degrees due to their newly acquired surfactant like properties (Figure 2).

Interestingly it was observed that oils that were not showing any QSI activity before, either alone or in combination with OASL, became potent inhibitors of quorum sensing mediated phenotypes upon transformation into their corresponding EOSLs (Table 1). Orange oil SL, citronella oil SL, and rosemary oil SL showed smaller zones of quorum sensing inhibition when compared to other EOSLs (Table 1); however individual oils showed no QSI activity. Also it was intriguing to note that all three EOSLs mentioned before with lesser QSI activity (orange oil SL, citronella oil SL, and rosemary oil SL) were found to be very similar in composition to OASL. Ylang ylang oil whose QSI potential has never been explored was used in this study for the first time and its EOSL has been shown to a very powerful inhibitor of QS mediated phenotype. Ylang ylang oil is extracted from fresh flowers of the tree of the same name, by water or steam distillation. It has many therapeutic properties like antidepressant, antiseborrheic, antiseptic, and hypotensive and EOSL of this oil with potent anti-QS activity will definitely have a broader range of medical application. Also other EOs used in this study whose QSI potential is being explored for the first time include bergamot oil, Frankincense oil, basil oil and tea tree oil. All these oils when used alone showed no QSI activity; however their EOSLs could very well inhibit QS mediated phenotypes. The order of QSI potential of EOSLs of the above-mentioned EOs is as follows: basil oil > tea tree oil > Frankincense oil = Bergamot oil (Table 1).

**Figure 3:**
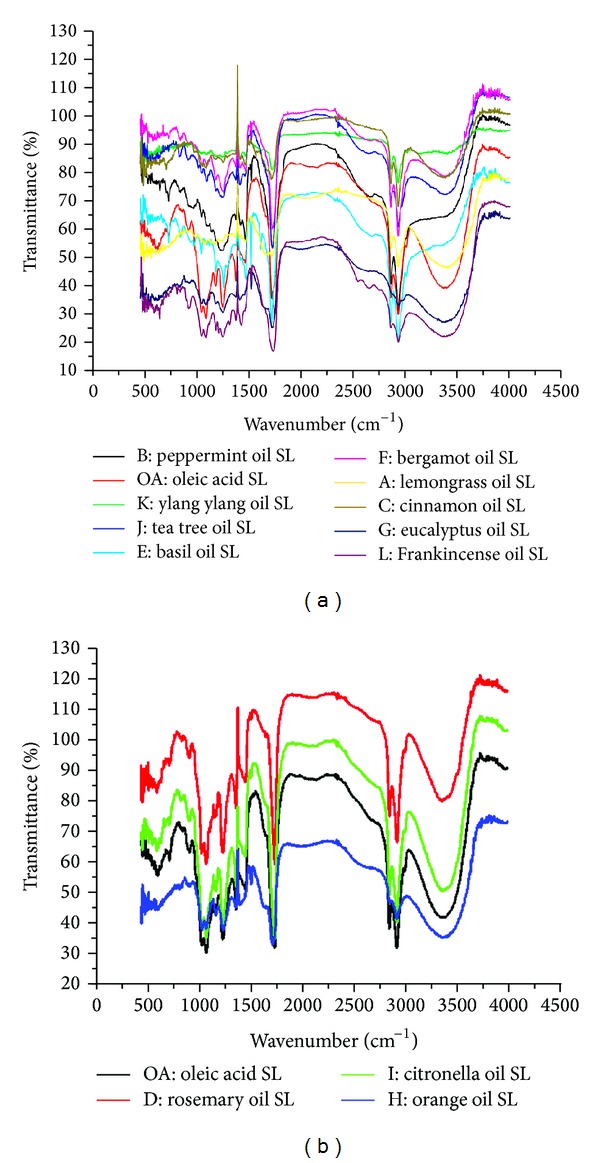
(a) FTIR analysis of the synthesized EOSLs along with OASL. (b) FTIR spectra of EOSLs with composition similar to OASL.

FTIR analysis demonstrated that synthesized EOSLs show certain peaks similar to those of OASL; however, certain new peaks could be seen in the spectra of the synthesized EOSLs. Aromatic ring structure C=C stretch around 1520–1515 cm^−1^, which may be arising from certain aromatic components of each essential oils, was observed in the spectra for basil oil SL, tea tree oil SL, Bergamot oil SL, eucalyptus oil SL, and Frankincense oil SL (Figure 3(a)). Peaks around 1760–1670 cm^−1^ arising from C=O of aldehydic, ketonic ester or carboxylic residues were seen in the spectra of lemongrass oil SL, cinnamon oil SL, basil, Bergamot, and Ylang ylang oil SL. Vibrational peaks specific to methylene groups (2850 and 2925 cm^−1^) and aromatic C–H bond (700–750 cm^−1^) were seen in the spectra of all oils; however the former peaks (specific to methylene groups) were also visible in OASL spectra (Figure 3(a)). C–C bond conjugated with benzene ring stretch (1600–1625 cm^−1^) was observed in the spectra of basil oil SL, eucalyptus oil SL, and Ylang ylang oil SL. Citronella, rosemary, and orange oil SLs showed spectra very similar to those of OASL and likewise their QSI activity was also not very pronounced (Figure 3(b)).

**Figure 4:**
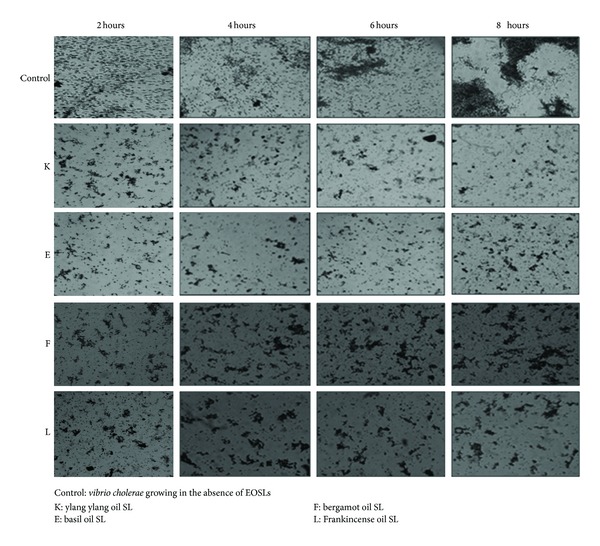
Antibiofilm activity of selected EOSLs.

Essential oils of lavender, tea tree, and lemon balm have been shown to have anti-biofilm activity using *Staphylococcus aureus* and *Escherichia coli* as test organism [22]. Also Schillaci et al. [23] have shown that essential oils from two *Bowellia *sp. (Frankincense oil) have the ability to inhibit biofilm formed by two species of *Staphylococcus* and *Candida albicans*. Peppermint oil has also been shown to inhibit biofilm formed by *C. albicans *[24]. Adukwu et al. [25] have shown anti-biofilm activity of lemongrass EO and grapefruit EO against five strains of* Staphylococcus aureus. *In our study antibiofilm activity of selected EOSLs but not EOs have been analyzed as they are expected to have improved ability to inhibit initial adhesion of microorganisms to solid surface due to their biosurfactant like property in addition to their antimicrobial and quorum sensing inhibitory property. Selected EOSLs were able to inhibit adhesion of microorganism to the glass surface and arrest biofilm formation in the initial stages itself (Figure 4). QSI potential of these EOSLs has been established before and that may be responsible for the obvious decrease in bacterial biofilm, because genes required for exopolysaccharide (EPS) production (EPS is the essential component for establishment of biofilm architecture and maturation [10]) are under QS control. Ylang ylang oil SL and basil oil SL both showed potent anti-biofilm activity as observed microscopically. Moreover test organism used in our study was *V. cholerae* which has added significance from an Indian subcontinent perspective because cholera is an endemic problem and biofilm formed by *V. cholerae* is an important part of its pathogenesis and disease establishment.

## 4. Conclusion

This paper highlights the advantage of conversion of EOs to EOSLs because this conversion reaction bestows additional chemical and physical properties to the component EOs making them better quorum sensing inhibitors and powerful anti-biofilm agents. Moreover the paper reports use of some EOs whose ability to inhibit quorum sensing has not been explored before, namely, Ylang ylang oil, Frankincense oil, basil oil, Bergamot oil, tea tree oil, and successful production of their EOSLs with enhanced QSI activity. Detailed characterization of all the synthesized EOSLs using LC-MS and HR-MS is underway. Further due to enhanced quorum sensing inhibitory and biosurfactant like property warrant the use of these EOSLs in topical formulation like hand washes which would aim to prevent spread of various communicable diseases.

## Figures and Tables

**Table 1:** Tabulated data showing quorum sensing inhibitory (QSI) activity of essential oils used in this study, alone and in combination with 10 mg/mL of OASL. Also QSI activity of all synthesized EOSLs.

Plant essential oil (common name)	Plant essential oil (scientific name)	Main component	Anti-QSI activity of essential oil alone (20% oil) against CV026 (inhibition zone size in mm)	Anti-QSI activity of EO + OASL against CV026 (inhibition zone size in mm)^a^	Anti-QSI activity of EOSL against CV026 (inhibition zone size in mm)^b^
Lemongrass oil	*Cymbopogon citratus *	Citral	15	28	25
Peppermint oil	*Mentha piperita *	Menthol	—	16	13
Cinnamon oil	*Cinnamomum verum *	Cinnamaldehyde	15 (10% EO)	20 (10% EO + OASL at 10 mg/mL)	Growth inhibition at 10 mg/mL EOSL.
Rosemary oil	*Rosmarinus officinalis *	(+) Alpha pinene	—	—	13
Basil oil	*Ocimum basilicum *	L-Linalool	—	11	30
Bergamot oil	*Citrus bergamia *	L-Linalool	—	—	17
Eucalyptus oil	*Eucalyptus sp. *	1,8-Cineole	—	—	23
Orange oil	*Citrus sinensis *	Limonene	—	—	13
Citronella oil	*Cymbopogon nardus *	Citronellal	—	—	12
Tea tree oil	*Melaleuca alternifolia *	Alpha terpineol	—	—	26
Ylang ylang oil	*Cananga odorata *	L-Linalool	—	12	33
Frankincense oil	*Boswellia carteri *	(+) Alpha pinene	—	—	17

^a^1 mL reaction mixture contained 20% EO and OASL at a concentration of 10 mg/mL. Also OASL alone used at a concentration of 10 mg/mL had no QSI activity.

^b^EOSL concentration used 20 mg/mL.

## References

[1] Bassolé IHN, Juliani HR (2012). Essential oils in combination and their antimicrobial properties. *Molecules*.

[2] Jaramillo-Colorado B, Olivero-Verbel J, Stashenko EE, Wagner-Dobler I, Kunze B (2012). Anti-quorum sensing activity of essential oils from Colombian plants. *Natural Product Research*.

[3] Kalemba D, Matla M, Smetek A (2012). Antimicrobial activities of essential oils. *Dietary Phytochemicals and Microbes*.

[4] El Moussaoui N, Sanchez G, Khay EO (2013). Antibacterial and antiviral activities of essential oils of Northern Moroccan plants. *British Biotechnology Journal*.

[5] Faleiro ML, Miguel MG, Ladeiro F (2003). Antimicrobial activity of essential oils isolated from Portuguese endemic species of *Thymus*. *Letters in Applied Microbiology*.

[6] Angioni A, Barra A, Coroneo V, Dessi S, Cabras P (2006). Chemical composition, seasonal variability, and antifungal activity of *Lavandula stoechas* L. ssp. *stoechas* essential oils from stem/leaves and flowers. *Journal of Agricultural and Food Chemistry*.

[7] Krishnan T, Yin W-F, Chan K-G (2012). Inhibition of quorum sensing-controlled virulence factor production in *Pseudomonas aeruginosa* PAO1 by ayurveda spice clove (*Syzygium aromaticum*) bud extract. *Sensors*.

[8] Chong YM, Yin WF, Ho CY (2011). Malabaricone C from myristica cinnamomea exhibits anti-quorum sensing activity. *Journal of Natural Products*.

[9] Zahin M, Hasan S, Aquil F, Khan MSA, Husain FM, Ahmad I (2010). Screening of certain medicinal plants from India for their anti-quorum sensing activity. *Indian Journal of Experimental Biology*.

[10] Abraham I, Packiavathy SV, Agilandeswari P, Musthafa KS, Pandian SK, Ravi AV (2012). Antibiofilm and quorum sensing inhibitory potential of *Cuminum cyminum* and its secondary metabolite methyl eugenol against Gram negative bacterial pathogens. *Food Research International*.

[11] Szabó MA, Varga GZ, Hohmann J (2010). Inhibition of quorum-sensing signals by essential oils. *Phytotherapy Research*.

[12] Khan MSA, Zahin M, Hasan S, Husain FM, Ahmad I (2009). Inhibition of quorum sensing regulated bacterial functions by plant essential oils with special reference to clove oil. *Letters in Applied Microbiology*.

[13] Bassler BL Small talk. Cell-to-cell communication in bacteria. *Cell*.

[14] Fuqua WC, Winans SC, Greenberg EP (1994). Quorum sensing in bacteria: the LuxR-LuxI family of cell density- responsive transcriptional regulators. *Journal of Bacteriology*.

[15] Joint I, Allan DJ, Williams P Bacterial conversations: talking, listening and eavesdropping. An introduction. *Philosophical Transactions of the Royal Society B*.

[16] Williams P, Winzer K, Chan WC, Cámara M (2007). Look who's talking: communication and quorum sensing in the bacterial world. *Philosophical Transactions of the Royal Society B*.

[17] Waters CM, Bassler BL (2005). Quorum sensing: cell-to-cell communication in bacteria. *Annual Review of Cell and Developmental Biology*.

[18] Williams P (2002). Quorum sensing: an emerging target for antibacterial chemotherapy?. *Expert Opinion on Therapeutic Targets*.

[19] Dengle-Pulate V, Bhagwat S, Prabhune A (2013). Microbial oxidation of medium chain fatty alcohol in the synthesis of sophorolipids by *Candida bombicola* and its physicochemical characterization. *Journal of Surfactants and Detergents*.

[20] Chu W, Vattem DA, Maitin V, Barnes MB, McLean RJC (2011). Bioassays of quorum sensing compounds using *Agrobacterium tumefaciens* and *Chromobacterium violaceum*. *Methods in Molecular Biology*.

[21] Nithya C, Pandian SK (2010). The *in vitro* antibiofilm activity of selected marine bacterial culture supernatants against *Vibrio* spp.. *Archives of Microbiology*.

[22] Budzyńska A, Wieckowska-Szakiel M, Sadowska B, Kalemba D, Rózalska B (2011). Antibiofilm activity of selected plant essential oils and their major components. *Polish Journal of Microbiology*.

[23] Schillaci D, Arizza V, Dayton T, Camarda L, Di Stefano V (2008). *In vitro* anti-biofilm activity of *Boswellia* spp. Oleogum resin essential oils. *Letters in Applied Microbiology*.

[24] Saharkhiz MJ, Motamedi M, Zomorodian K, Pakshir K, Miri R, Hemyari K (2012). Chemical composition, antifungal and antibiofilm activities of the essential oil of *Mentha piperita* L.. *ISRN Pharmaceutics*.

[25] Adukwu EC, Allen SCH, Phillips CA (2012). The anti-biofilm activity of lemongrass (*Cymbopogon flexuosus*) and grapefruit (*Citrus paradisi*) essential oils against five strains of *Staphylococcus aureus*. *Journal of Applied Microbiology*.

